# Health Information From Web Search Engines and Virtual Assistants About Pre-Exposure Prophylaxis for HIV Prevention in Adolescents and Young Adults: Content Analysis

**DOI:** 10.2196/41806

**Published:** 2023-07-18

**Authors:** Kaja Darien, Susan Lee, Kayla Knowles, Sarah Wood, Miriam D Langer, Nellie Lazar, Nadia Dowshen

**Affiliations:** 1 PolicyLab Children's Hospital of Philadelphia Roberts Center for Pediatric Research Philadelphia, PA United States; 2 Craig-Dalsimer Division of Adolescent Medicine Children's Hospital of Philadelphia Philadelphia, PA United States; 3 Department of Pediatrics Perelman School of Medicine University of Pennsylvania Philadelphia, PA United States

**Keywords:** pre-exposure prophylaxis, PrEP, prophylaxis, internet use, search engine, adolescent, youth, pediatric, adolescence, young adult, readability, human immunodeficiency virus, HIV, virtual assistant, health information, information quality, accuracy, credibility, patient education, comprehension, comprehensible, web-based, online information, sexual health, reading level

## Abstract

**Background:**

Adolescents and young adults are disproportionately affected by HIV, suggesting that HIV prevention methods such as pre-exposure prophylaxis (PrEP) should focus on this group as a priority. As digital natives, youth likely turn to internet resources regarding health topics they may not feel comfortable discussing with their medical providers. To optimize informed decision-making by adolescents and young adults most impacted by HIV, the information from internet searches should be educational, accurate, and readable.

**Objective:**

The aims of this study were to compare the accuracy of web-based PrEP information found using web search engines and virtual assistants, and to assess the readability of the resulting information.

**Methods:**

Adolescent HIV prevention clinical experts developed a list of 23 prevention-related questions that were posed to search engines (Ask.com, Bing, Google, and Yahoo) and virtual assistants (Amazon Alexa, Microsoft Cortana, Google Assistant, and Apple Siri). The first three results from search engines and virtual assistant web references, as well as virtual assistant verbal responses, were recorded and coded using a six-tier scale to assess the quality of information produced. The results were also entered in a web-based tool determining readability using the Flesch-Kincaid Grade Level scale.

**Results:**

Google web search engine and Google Assistant more frequently produced PrEP information of higher quality than the other search engines and virtual assistants with scores ranging from 3.4 to 3.7 and 2.8 to 3.3, respectively. Additionally, the resulting information generally was presented in language at a seventh and 10th grade reading level according to the Flesch-Kincaid Grade Level scale.

**Conclusions:**

Adolescents and young adults are large consumers of technology and may experience discomfort discussing their sexual health with providers. It is important that efforts are made to ensure the information they receive about HIV prevention methods, and PrEP in particular, is comprehensive, comprehensible, and widely available.

## Introduction

The HIV epidemic is a major health concern, with the Centers for Disease Control and Prevention (CDC) reporting 37,698 new HIV diagnoses in the United States in 2018 [1,2]. While individuals aged 25-44 years had the highest incidence of HIV, youth aged 13-24 years comprised more than half of all new diagnoses [1].

Pre-exposure prophylaxis (PrEP) is an effective daily prevention method and should be considered for use by HIV-negative populations at higher risk of HIV [3]. When taken as prescribed, PrEP can offer almost complete protection against HIV; however, previous studies have shown that PrEP uptake is lower in higher risk populations [4,5]. Barriers to PrEP uptake include limited awareness and limited access as a result of PrEP marketing and additional socioeconomic challenges, respectively [5].

Moreover, adolescents and young adults are major consumers of technology and often rely on the internet for health information [1,6-8]. Since adolescents and young adults may be consulting the internet instead of health care providers for HIV prevention information, it is critical that health information on the web is accurate and readable. To the authors’ knowledge, there is limited research assessing the quality of PrEP information available on the web. Therefore, the objectives of this study are to determine if accurate and readable PrEP information can be found using web search engines and virtual assistants. Additionally, this study aimed to assess how PrEP information found via both methods compared among and between search engines and virtual assistants.

## Methods

### Ethical Considerations

The institutional review board at Children’s Hospital of Philadelphia determined that approval was not necessary for this study as it did not contain human subjects.

### Developing a Framework for Evaluating PrEP Internet Content

Common PrEP information themes were determined for future searches within the United States using English only: PrEP basics, PrEP access, and PrEP use (Textbox 1). A total of 50 questions were compiled through an iterative process with an expert panel of adolescent medicine researchers and physicians who work with adolescents and young adults at high risk of contracting HIV as well as youth living with HIV. Items were narrowed down to 23 questions designed to address concerns adolescents and young adults may have about general use, privacy, and confidentiality (Table 1).

**PrEP categories.**

**Pre-exposure prophylaxis (PrEP) basics**

- The questions in this category aim to find general information about PrEP for HIV prevention to introduce and educate potential users about this intervention method.

**PrEP access**

- The questions in this category aim to find information about ways users can access PrEP in addition to information about privacy concerns.

**PrEP use**

- The questions in this category aim to find information regarding the proper use of PrEP so that potential users can receive the maximum benefits.

**Table 1:** PrEP internet search questions.

Question	Category
What is PrEP^a^?	Basics
What kinds of PrEP are available?	Basics
What is Truvada?	Basics
What is Descovy?	Basics
What is the difference between Truvada and Descovy?	Basics
Does PrEP protect against other sexually transmitted infections/sexually transmitted diseases?^b^	Basics
Should I take PrEP?	Basics
What are the side effects of taking PrEP?	Basics, use
Will PrEP make me feel sick?	Basics
How does PrEP work?	Basics, use
What is the difference between PEP^c^ and PrEP?	Basics
How effective is PrEP?	Basics, use
Where can I find PrEP?	Access
How can I get PrEP?	Access
Do I need insurance to get PrEP?	Access
Do I need my parents’ permission to get PrEP?	Access
Will my parents know that I’m using PrEP if I’m on their insurance?	Access
How much does PrEP cost?	Access
How do I take PrEP?	Use
When can I stop taking PrEP?	Use
How long does it take for PrEP to start working?	Use
What happens if I miss a dose of PrEP?	Use, basics
How do I refill my PrEP prescription?	Use, access

^a^PrEP: pre-exposure prophylaxis.

^b^This question was asked using both “sexually transmitted infections” and “sexually transmitted diseases” due to the recent change in terminology.

^c^PEP: postexposure prophylaxis.

### Data Collection: Web Search Engines and Virtual Assistants

Questions were entered into search engines—Ask.com, Bing, Google, and Yahoo—using a private Mozilla Firefox browser and verbally posed to virtual assistants—Amazon Alexa, Microsoft Cortana, Google Assistant, and Apple Siri—using new user accounts apart from Apple Siri where a pre-existing account with cleared browsing history was used. Questions were asked to search engines and virtual assistants on separate days in March 2021 with a maximum of 10 times for virtual assistants to generate a response. Excluding advertised content, information from the first three web search engine results and virtual assistant nonverbal results were transcribed as listed on the website and recorded in corresponding Excel (Microsoft Corporation) sheets for future analysis. Verbal responses from each virtual assistant were recorded, transcribed, and saved in Excel.

### Data Analyses: Scale Development, Rating System for Information Accuracy, and Readability Assessment

A six-tier scale was created and reviewed by an adolescent medicine physician and a researcher to code example content (Table 2). The examples were used as content validation to ensure that results were coded using the same criteria. Results received two scores from two different coders who assigned final scores after reaching a consensus. Final scores were then used to calculate an average quality score for each web-based resource in each category. The data collected were also entered into a web-based literacy measurement tool [9] to assess readability using the Flesch-Kincaid Grade Level Scale [10].

**Table 2:** PrEP Information Quality Rating Scale.

Rating number	Meaning	Example content (question: what is PrEP^a^?)
0	The resulting answer did not include any information about PrEP.	“HIV (human immunodeficiency virus) is a virus that attacks the body’s immune system. There is currently no effective cure. Once people get HIV, they have it for life” [11]
1	The resulting answer included information about PrEP that was completely inaccurate.	“PrEP is a medication taken only right before sex in order to prevent HIV. While taking PrEP, individuals do not need to use condoms” [12]
2	The resulting information about PrEP was partially correct but contained some errors/misleading information. The source providing information was unreliable.	“PrEP is a pill taken before sex, so it is pre-exposure. Prophylaxis means to prevent infection. So you can use PrEP to greatly reduce the risk of becoming HIV positive” [13]
3	The resulting information was accurate but lacked many important details.	“Pre-exposure Prophylaxis, also known as PrEP, is when people take medicine to lower their risk of getting HIV” [14]
4	The resulting information was detailed, accurate, and from a reliable source. However, the information included was outdated.	“PrEP is an experimental approach to HIV prevention and consists of antiretroviral drugs to be taken before potential HIV exposure in order to reduce the risk of HIV infection and continued during periods of risk” [15]
5	The resulting information was detailed, accurate, and from a reliable source. Additionally, the result included the latest information available.	“Pre-exposure prophylaxis (or PrEP) is a way for people who do not have HIV but who are at very high risk of getting HIV to prevent HIV infection by taking a pill every day. The pill (brand name Truvada) contains two medicines (tenofovir and emtricitabine) that are used in combination with other medicines to treat HIV. When someone is exposed to HIV through sex or injection drug use, these medicines can work to keep the virus from establishing a permanent infection. When taken daily, PrEP is highly effective for preventing HIV. Studies have shown that PrEP reduces the risk of getting HIV from sex by about 99% when taken daily. Among people who inject drugs, PrEP reduces the risk of getting HIV by at least 74% when taken daily. PrEP is much less effective if it is not taken consistently. As PrEP only protects against HIV, condoms are important for the protection against other STDs. Condoms are also an important prevention strategy if PrEP is not taken consistently.” [16]

^a^PrEP: pre-exposure prophylaxis.

## Results

### Web-Based PrEP Information Quality

From all search engines and virtual assistants, 422 results were compiled and assigned a score from the PrEP Information Quality Scale (Table 2). The results show that Google search engine and Google Assistant more frequently provided higher quality information than resources from other companies, and that search engines provided higher quality information than virtual assistants (Table 3). Google search engine provided more comprehensive results directing potential users to websites with information on the ideal candidates, proper use, and access. However, Ask.com often provided the lowest quality information directing users to websites containing no information about PrEP. Bing and Yahoo provided information quality between Google and Ask.com.

**Table 3:** Search engines’ and virtual assistants’ average PrEP information quality scores^a^.

Resource			PrEP^b^ basics		PrEP access		PrEP use
**Search engines**							
	Ask.com	1.8		0.8		0.5	
	Bing	3.7		2.7		3.1	
	Google	3.4		3.7		3.5	
	Yahoo	3.4		2.8		2.9	
**Virtual assistants**							
	Amazon Alexa	1.1		0.9		1.4	
	Apple Siri	2.4		1.8		3.3	
	Google Assistant	3.2		3.3		2.8	
	Microsoft Cortana	2.3		2.5		2.0	

^a^PrEP information score meanings: 0=irrelevant, 1=inaccurate, 2=partially correct/misleading, 3=accurate but missing important details, 4=accurate but outdated, 5=accurate and current.

^b^PrEP: pre-exposure prophylaxis.

The following responses were given to the question “How much does PrEP cost?”

...A month’s supply of Truvada is nearly $2,000 without insurance. Most private health insurance companies, Medicare, and Medicaid will cover the cost...

WebMD via Google

Launching a startup...

Ask.com

Within virtual assistants, Google Assistant typically provided more informative results when compared to the other virtual assistants selecting relevant text from websites to respond to posed questions. Amazon Alexa generally provided the lowest quality information with irrelevant responses including “Sorry, I don’t know that one.” Microsoft Cortana and Apple Siri provided information quality between Google Assistant and Amazon Alexa.

The following responses were given to the question “What is the difference between Truvada and Descovy?”

There are now two medications approved by the U.S Food and Drug Administration (FDA): Truvada and Descovy...which medication might be right for you, take a look at the similarities and differences between the two medications below...

SFAF.org via Google Assistant

Pharmaceutical product. Star Trek Discovery...

Amazon Alexa

### Web-Based PrEP Information Readability

The readability assessment using the Flesch-Kincaid Grade Level scale revealed that information from web search engines produced by questions in all three categories requires a literacy level that aligns with that of an eighth grade, ninth grade, or 10th grade student, while virtual assistant results require literacy levels ranging from a second grade to eighth grade student in the United States (Table 4). The required literacy level of information produced by search engines and virtual assistants appears to correlate with the quality of information produced by each, as the search engines more frequently provided information of higher quality than the virtual assistants.

**Table 4:** Search engines’ and virtual assistants’ average PrEP information readability scores^a^.

Resource		PrEP^b^ basics		PrEP access		PrEP use
**Search engines**						
	Ask.com	8.3	9.2		8.3	
	Bing	10.2	8.1		9.1	
	Google	9.3	9.6		8.6	
	Yahoo	9.7	8.1		9.2	
**Virtual assistants**						
	Amazon Alexa	7.0	2.7		5.4	
	Apple Siri	8.4	4.8		7.1	
	Google Assistant	7.4	8.3		6.9	
	Microsoft Cortana	7.0	6.4		7.7	

^a^PrEP information readability score formula: grade level = 0.37 (words/sentence) + 5.84 (syllables/word) – 15.59.

^b^PrEP: pre-exposure prophylaxis.

## Discussion

### Principal Results

Google search engine and Google Assistant produced higher quality PrEP information more frequently than the other web-based resources used. Authors found resulting answers to PrEP questions that conflicted with each other within a single search engine/virtual assistant, which may lead to incorrect use of PrEP and ultimately reduced effectiveness. Additionally, the resulting information generally was presented in language between a seventh and 10th grade reading level, therefore, often exceeding the average reading level of adolescents and young adults in the United States [17].

### Comparison With Prior Work

To the authors’ knowledge, there are no other studies that have specifically evaluated the quality of PrEP information for HIV prevention produced by virtual assistants or the readability of health information found on the web. However, a New Zealand study titled “In Bed with Siri and Google Assistant: A Comparison of Sexual Health Advice” used Google search engine, Google Assistant, and Apple Siri to assess the quality of sexual health advice received from each source. The results of this study are consistent with researchers’ findings that Google search engine had the best responses, followed by Google Assistant then Apple Siri, when asked questions about sex and sexual health [18].

### Limitations

The study limitations include the variability of results produced by search engine and virtual assistant algorithms. To account for the possibility of different top results for the same questions, searches were performed on a single day for all search engines and on a separate day within the same week for all virtual assistants. Future studies may consider conducting a time-based analysis where searches are completed at set time intervals over 1 year or more to compare top results for the same questions over time. Another limitation is the condensed list of questions. Future studies should include youth perspectives on what questions adolescents and young adults would ask in addition to provider opinions. Additionally, the study was conducted in English, which may exclude individuals whose primary language is not English. Furthermore, at the time of this study, injectable PrEP had not yet been approved and are not included in the search results.

### Conclusions

Adolescents and young adults may turn to technology due to discomfort discussing their sexual health with providers, yet we found that there is much room for improvement in the quality and readability of educational information on PrEP through web-based resources.

## Abbreviations

CDC: Centers for Disease Control and Prevention
PrEP: pre-exposure prophylaxis
